# Strike Out: A Case Report of Glioblastoma in a Collegiate Softball Player

**DOI:** 10.7759/cureus.44486

**Published:** 2023-08-31

**Authors:** Rock P Vomer, Dusty Narducci, Rayghan S Larick, Emma York, Kristin Terry

**Affiliations:** 1 Department of Family Medicine, Mayo Clinic, Jacksonville, USA; 2 Department of Family Medicine, Avance Care, Raleigh, USA; 3 Department of Family Medicine, University of South Florida Morsani College of Medicine, Tampa, USA; 4 Department of Family and Community Medicine, Eastern Virginia Medical School, Norfolk, USA; 5 Department of Family Medicine and Sports Medicine, University of South Carolina, Columbia, USA

**Keywords:** sports participation, neurology and neuro-oncology, athlete, pre-participation examinations, malignancy, glioblastoma

## Abstract

A 20-year-old female, right-hand-dominant Division I softball player, presented to her pre-participation exam endorsing numbness that started in her left thumb and had progressed to involve her entire hand and left medial elbow. She had no change in her physical health over the past year prior to presentation and denied injury illness or trauma to the left upper extremity or neck. She reported no change in her softball off-season training regimen or equipment. Exam exhibited decreased sensation in C6, C7, and C8 dermatomes and weakness in the C8 myotome. Magnetic resonance angiography (MRA) displayed a right parietal lobe mass which biopsy confirmed as glioblastoma multiforme (GBM). GBM, also known as glioblastoma or grade 4 astrocytoma is an aggressive form of cancer that can affect the brain and spinal cord. Despite being the most common malignant primary brain tumor in adults, current treatment is mostly palliative. Treatment for this student-athlete included surgery, radiation, and chemotherapy. The selection of aggressive treatment including excision of the tumor was largely elected due to her age. She received chemotherapy with temozolomide in conjunction with radiation for a total of nine months. Following treatment, she worked with physical therapy to help improve her functional deficits, caused both by the tumor as well as the excision. Additionally, psychological and emotional support was provided to the patient during the course of the diagnosis and treatment of her athletics career-ending diagnosis. The same support services were also extended to the entirety of her teammates as well as her family members. This case outlines the diagnosis, treatment, and challenges of GBM in a Division I softball athlete including the challenges of providing emotional support for an athlete living away from home while being diagnosed with a life and athletics-career altering condition.

## Introduction

Glioblastoma multiforme (GBM), also known as glioblastoma or grade 4 astrocytoma, is a highly aggressive tumor of glial cells and is the most common malignant primary brain tumor in adults [1]. In the United States, the incidence of GBM is 3.2/100,000 in the general population and its incidence increases to 15.24/100,000 between age 75 to 84 years [2]. There is a slightly higher incidence in men compared to women (1.6:1) [3]. The overwhelming majority of glioblastoma diagnoses have no known familial predilection and fewer than 1% of glioblastomas are attributed to genetic predisposition associated with diseases such as neurofibromatosis 1 and 2, retinoblastoma, tuberous sclerosis, Turcot’s syndrome, and Li-Fraumeni [3]. The only known risk factor that has shown definitive association with the development of brain tumors, including glioblastoma, is ionizing radiation [1,3].

GBM typically presents with functional impairments corresponding to the location of central nervous system involvement [1,2]. Common presenting symptoms include signs of increased intracranial pressure including headache, sensory changes including numbness and loss of vision, language difficulties, changes in memory, and persistent weakness [1,2]. Seizures occur as a presenting symptom in approximately 25% of patients [2]. Diagnosis of GBM relies on magnetic resonance (MR) followed by biopsy or resection with histopathology confirmation [2]. 

Despite recent advances in the treatment of GBM, it remains very difficult to treat and carries a generally poor prognosis with a mean five-year survival of 5% [2]. Poor prognosis is related to difficulty in limited effectiveness of current treatment options, including difficulty in achieving complete surgical resection of tumor coupled with high rates of recurrence [3,4]. Treatment involves a multifactorial approach with surgical resection, radiotherapy, and chemotherapy. More recently, with the emphasis of precision medicine, novel treatments have been developed to directly target tumors based on their genetic and molecular features. Current standard treatment involves maximal safe surgical resection followed by postoperative chemoradiation therapy and adjuvant chemotherapy, most commonly with temozolomide (TMZ) an oral chemotherapeutic methylating agent [1,3,5]. 

This report outlines the diagnosis and treatment of GBM in a Division I softball athlete and highlights the unique circumstances associated with the management of the condition in this patient population. We aim to outline the clinical approach to the diagnosis of GBM and describe current treatment options and their limitations. Although a diagnosis of high-grade glioblastoma in a collegiate athlete is rare, this case provides learning opportunities for the field of sports and exercise medicine.

## Case presentation

History

A 20-year-old female Division I right-handed softball athlete presented for a routine returning pre-participation examination (PPE). Her past medical history was significant for attention deficit hyperactivity disorder (ADHD), which was controlled by taking methylphenidate. Her only other medication was a combined oral contraceptive. She had reported no change in her physical health over the last 12 months prior to her PPE. At the time of evaluation, the patient reported numbness that started in her left thumb and progressed to involve her entire hand and left medial elbow over the course of the summer without any other symptoms. She denied injury illness or trauma to the left upper extremity or neck. She reported no change in her softball off-season training regimen or equipment.

Physical exam

Vital signs were within normal limits and the patient appeared well-nourished and had an athletic build. The vision was grossly intact. Lungs clear to auscultation bilaterally. Cardiac exam revealed a regular rate and rhythm with no murmurs, rubs, or gallops appreciated. The abdomen was soft and non-tender with no palpable masses. There were no visible skin lesions or rashes. No lymphadenopathy was appreciated in the cervical or axillary regions. The athlete displayed a normal mood and affect.

Examination of the left upper extremity revealed no obvious deformity, atrophy, induration, or erythema. Palpation slightly distal to the left medial epicondyle elicited mild tenderness. There was a full and equal active range of motion for the cervical spine, hand, wrist elbow, and shoulder. Special tests including Tinel’s at the cubital tunnel and wrist, Phalen’s, and Spurling’s maneuver were negative. Functional testing of the wrist and hand demonstrated age-appropriate fine motor skills and adequate hand strength to grasp and manipulate small objects.

Sensory examination demonstrated decreased sensation to light touch in the cervical 6, 7, and 8 dermatomes. The patient’s myotome testing revealed slight weakness in the cervical 8 dermatome. There were no changes in strength or sensation in the right upper extremity or bilateral lower extremities.

Differential diagnosis

The differential diagnosis for this case was broad given the patient's presentation. The initial differential diagnosis included the following: cervical nerve root disorder, median nerve entrapment, interosseous nerve entrapment, cerebral vascular accident, multiple sclerosis (MS), and brain tumor.

Cervical radiculopathy can cause pain, numbness, tingling, and weakness in the upper extremity, and location on the upper extremity depends on the cervical level involved. The patient had no neck pain or reproduction of symptoms with Spurling's during the exam. Median nerve entrapment often causes numbness in the first three digits and paresthesia. Symptoms can mimic injury to the cervical 6 and 7 nerve roots. The patient's symptoms did not involve any digits other than her thumb. Phalen's and Tinel's tests at the wrist during examination were also negative. Similarly, interosseous nerve entrapment causes deficits in sensation where the nerve provides innervation. Our patient also demonstrated C8 myotome weakness making an interosseous entrapment less likely. A cerebral vascular accident was considered as she was at higher risk for a vaso-occlusive event being on an oral contraceptive pill. A frontoparietal infarction could lead to the deficits displayed by the athlete; however, without a personal history or family history of prior coagulopathy, the likelihood of a cerebrovascular accident (CVA) in a 20-year-old was unlikely.

MS was also considered given MS's predilection for being diagnosed in young females in their 20s. However, typically symptoms wax and wane, unlike our patient's symptoms which not only remained present but were also progressive. The location of the brain lesions associated with MS corresponds to the symptoms it presents, which can include change in vision, numbness, tingling, or muscle contraction difficulties among others.

Brain tumor at the time of evaluation was considered unlikely given the low number of brain tumors diagnosed annually, the majority of tumors being diagnosed in elderly populations, and no presence of other commonly reported symptoms such as headache, change in vision, nausea, vomiting, or seizure activity. 

Imaging/diagnostics

**Figure 1:**
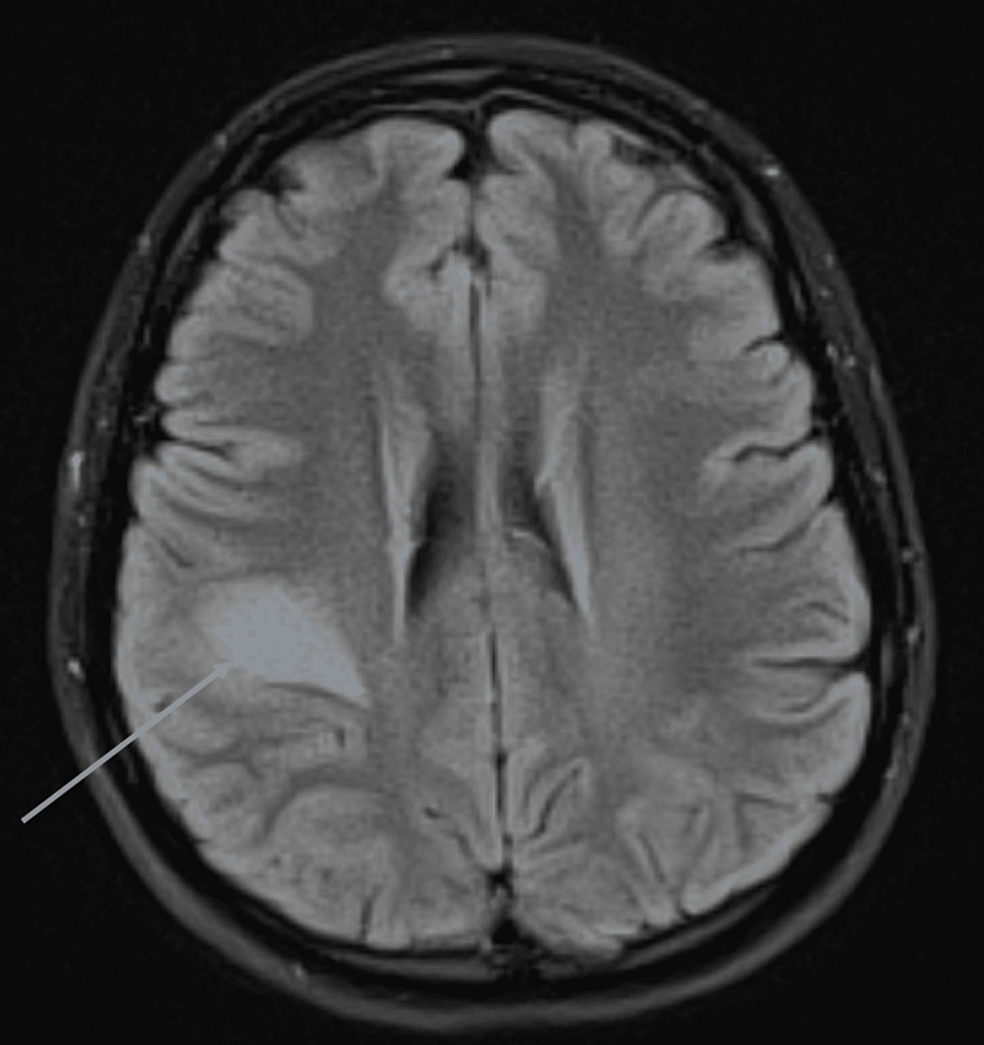
Brain MRI with contrast demonstrating a subacute ride-sided frontal parietal infarction (arrow), exhibiting diffusion restriction, FLAIR, DWI hyperintensity FLAIR, fluid-attenuated inversion recovery; DWI, diffusion-weighted imaging

Prior cervical radiographs demonstrated no abnormal findings. Electromyography (EMG) of the left upper extremity revealed no evidence of left-arm mono or polyneuropathy, brachial plexopathy, or cervical radiculopathy (Table 1). Given the athlete’s progressive upper extremity symptoms, brain MR imaging (MRI) with and without contrast was performed for further assessment. The brain MRI revealed a subacute right-sided fronto-parietal infarction, exhibiting diffusion restriction, fluid-attenuated inversion recovery (FLAIR), and diffusion-weighted imaging (DWI) hyperintensity (Figure 1).

**Table 1:** EMG of left upper extremity demonstrating no evidence of left-arm mono or polyneuropathy, brachial plexopathy, or cervical radiculopathy Rel, Relative; APB, abductor pollicis brevis; ADM, abductor digiti minimi; Lat, latency; Diff, difference; EMG, electromyography

Motor Nerve Conduction:									
Nerve/Sites	Muscle	Latency ms	Amplitude mV	Rel Amp %	Duration ms	Segments	Distance mm	Lat Diff ms	Velocity m/s
L Median - APB									
Wrist	APB	2.56	14.7	100	6.38	Wrist – APB	65		
Elbow	APB	6.06	14.8	101	6.40	Elbow – Wrist	220	3.50	63
L Ulnar – ADM									
Wrist	ADM	2.65	13.5	100	6.35	Wrist – ADM	65		25
B. Elbow	ADM	5.73	12.9	95.7	6.75	B. Elbow - Wrist	185	3.08	60
A. Elbow	ADM	7.19	13.5	104	6.54	A. Elbow – B. Elbow	110	1.46	75
Sensory Nerve Conduction:									
Nerve/Sites	Rec. Site	Onset Lat ms	Peak Lat ms	O.P. Amp mV	P.P Amp mV	Segments	Distance mm	Peak Diff ms	Velocity m/s
L Median, Ulnar – Transcarpal comparison									
Median Palm	Wrist	1.44	1.90	38.7	46.9	Median Palm - Wrist	80		56
Ulnar Palm	Wrist	1.44	1.90	17.5	15.7	Ulnar Palm - Wrist	80		56
						Median Palm – Ulnar Palm		0.00	
L Radial – Anatomincal snuff box (Forearm)									
Forearm	Wrist	1.54	2.23	31.7	39.7	Forearm – Wrist	100		65
L Lateral antebrachial cutaneous – Forearm (Elbow)									
Elbow	Forearm	1.60	2.27	8.4	4.0	Elbow – Forearm	120		75
L Medial antebrachial cutaneous – Forearm (Elbow)									
Elbow	Forearm	1.83	2.40	19.8	14.5	Elbow – Forearm	120		65

The athlete was immediately transferred to the university-affiliated trauma hospital. At the emergency room, the athlete was placed on aspirin, and she was evaluated by the stroke team. Her coagulopathy profile was negative. Echocardiography was negative for thrombus, valvular abnormalities and patent foramen ovale. MR angiography (MRA) of the carotids demonstrated no significant hemodynamic stenosis.

**Figure 2:**
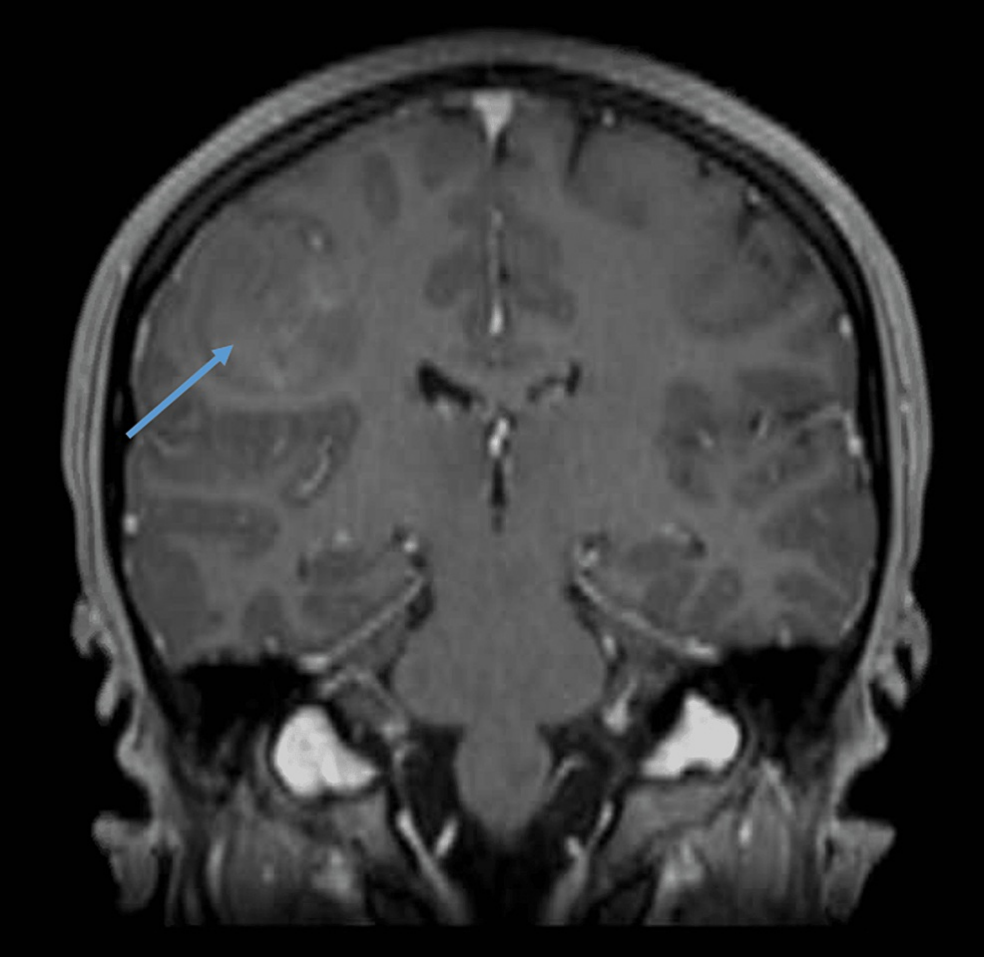
MRA Brain without contrast demonstrating a mass (arrow) in the right parietal lobe measuring approximately 4x3x3 cm

She was discharged from the emergency room with recommendations to take aspirin daily, discontinue her oral contraceptive pill, and follow up with neurology and cardiology outpatient. She was held from sports participation at this time. Over the next few days, she reported progressive, more pronounced upper extremity paresthesias and weakness. A MRA brain was consequently ordered, which displayed a mass in the right parietal lobe measuring approximately 4x3x3 cm with decreased T1 and subtle patchy enhancement, suggestive of a primary tumor (Figure 2). She was referred to oncology at a world-renowned cancer institute. Next, a stereotactic biopsy was performed to obtain a histologic diagnosis. The surgical pathology report of the mass revealed the diagnosis of GBM and was positive for a mutation in EGFR, MGMT promoter methylation, and negative for IDH, TERT promoter.

Treatment

The final diagnosis was a high-grade glioblastoma positive for mutation in EGFR, MGMT promoter methylation of the right parietal lobe as confirmed by histopathology during stereotactic biopsy. The treatment plan was formulated following this report by a team consisting of neuro-oncology and neurosurgery and followed the current gold-standard approach for GBM: surgical resection, radiation, and chemotherapy. 

The brain tumor was excised prior to radiation and chemotherapy. Excision is the principal element of GBM treatment as it often reverses symptoms that are present at the time of diagnosis, can control seizure activity, provides an opportunity for local placement of therapeutic agents, and overall can help improve the patient's quality of life [6]. GBM cannot be cured by excision alone due to the invasive nature of the tumor cells and the high likelihood of recurrence within a 2-3cm margin of the excision of the original tumor. Due to this, GBM reoccurs in upwards of 80% of cases following excision [7,8]. While excision is typically performed, it is location dependent. If GBM presents in the brain stem, for instance, surgical resection is not feasible. Given the location of our patient's tumor in the parietal lobe, she was able to undergo complete excision [9]. 

Radiation following surgical resection is commonly performed in an attempt to destroy any remaining cancer cells that may not have been excised. Radiation has been shown to improve life expectancy following resection in those with high-grade gliomas; thus, it is a staple in GBM treatment [9]. Radiation therapy unfortunately has many associated risks in addition to its benefits including radiation necrosis and permanent surrounding neuronal damage [9]. Our patient underwent radiation treatment following excision concomitantly with TMZ chemotherapy for six weeks without significant side effects from radiation therapy. Administration of chemotherapy during the radiation period is often chosen due to TMZ's ability to sensitize cells to radiation therapy increasing the likelihood of radiation-inducing cell death of the cancer cells [10]. 

She underwent further adjuvant chemotherapy treatment following these six weeks with TMZ for three additional rounds of six weeks each followed by a three-week hiatus. TMZ, an alkylating agent, is currently the standard chemotherapy agent used against GBM. Other alkylating agents that have been used previously have been shown to produce early cell resistance and have unfavorable side effect profiles, making TMZ the only effective alkylating chemotherapy agent [9]. TMZ achieves cancer cell death by forming 6-methylguanine lesions [10]. One of the difficulties with GBM treatment is its largely resistant nature to radiation and chemotherapy. Additionally, there are an inherently low number of treatment options due to the requirement that chemotherapy agents be able to cross the blood-brain barrier to reach the tumor cells [10,11]. 

As the patient received chemotherapy, she was prescribed anti-convulsant, anti-emetic, and medications to assist with pain. She also continued on her stimulant medication for ADHD. As the patient was considered a student-athlete, her school team physician still provided support and primary care throughout treatment. Additionally, she had access to additional services through the athletics department including physical therapy and psychology services, which were able to be initiated immediately upon diagnosis for mental and physical well-being. Physical therapy and/or occupational therapy are typically included in treatment to help patients with deficits either caused by the location of the tumor itself or after excision.

Psychology and emotional support resources were additionally provided to her family and teammates through the school's services. The team physicians, sports psychologist, certified athletic trainers, and coaching staff provided continuous resources to all student-athletes and university athletic personnel affected by the situation. 

Outcome

Due to diagnosis and treatment requirements, the athlete did not return to competitive softball and was required to take a medical leave of absence from school initially. She was able to return to school where she is still a student today and continues to receive care from her team primary care physician. At this time, two years later, her GBM treatment is ongoing with continued palliative chemotherapy and radiation. 

## Discussion

This report outlines the diagnosis and treatment of a 20-year-old softball player who was diagnosed with GBM. She presented to her PPE for the upcoming school year endorsing new numbness in her left hand without a history of recent trauma, injury, or changes to her training or equipment. Her physical exam displayed decreased sensation to light touch in the cervical 6, 7, and 8 dermatomes and slight weakness in the cervical 8 dermatome. Due to the presenting symptoms, she underwent extensive testing which included negative cervical spine x-rays, negative EMG, abnormal MRI brain scan concerning for CVA, negative MRA of the carotids, and a negative echocardiogram. She was subsequently treated for a CVA and follow-ups with neurology and cardiology were scheduled. However, prior to follow-up, her upper extremity numbness continued to worsen prompting repeat brain imaging with an MRA which demonstrated a mass in the right parietal lobe. A stereotactic biopsy of the mass confirmed the diagnosis of GBM. The athlete then was treated by her neuro-oncology team with surgical resection of the mass followed by radiation and chemotherapy. Back at the university, the athlete received continued support and treatment through the school’s physical therapists, psychologists, and team doctors as well as support from her teammates, coaching staff, and athletic training staff. Unfortunately, due to the nature of her diagnosis, she was unable to continue her softball career and had to take a medical leave of absence from the university while undergoing extensive treatment following her diagnosis. Eventually, she was able to return to her university as a student while continuing care under the guidance of both her neuro-oncology team as well as her former team doctors. 

Although a diagnosis of high-grade glioblastoma in a collegiate athlete is rare given the median age at diagnosis in the United States of 55-60 years, this case provides learning opportunities for the field of sports and exercise medicine [3]. The presentation of progressive, nontraumatic upper extremity neurologic symptoms in a collegiate softball athlete is most typically found to be a result of cervical nerve root disorder, ulnar or median nerve entrapment, brachial plexopathy, or interosseous nerve entrapment. While less likely, CVA, MS, hereditary neuropathy, Guillain-Barré syndrome variant, and brain tumors are diagnoses to keep in mind. Consideration of a wide differential upon presentation of symptoms similar to our athlete is important for appropriate and timely diagnosis. 

Given the presentation, nerve studies were conducted first with EMG. In this case, her normal EMG findings made neuropathy and similar etiologies less likely and redirected the cause of this athlete’s symptoms toward a cerebral etiology. Though an EMG was not of significant diagnostic value in this case, clinicians need to be aware that EMGs conducted earlier than four to six weeks following the onset of symptoms may provide a false negative result [5]. The location of the athlete’s left upper extremity numbness and paresthesia was comparable to Penfield and Rasmussen’s first map of cortical homunculus. The athlete’s brain mass compressed the primary somatosensory area in the parietal lobe of the cerebral cortex causing paresthesia from her left distal (thumb) to proximal (elbow). If compression, due to mass effect, or other compromise to the cerebral cortex occurs, signs and symptoms including aphasia, neglect, and/or cortical sensory loss are common [4]. This athlete’s brain MRI with and without contrast demonstrated a subacute ride-sided frontal parietal infarction with no pre or post-contrast imaging suggestive of abnormally enhancing brain lesions. One week later, an MRA of the brain without contrast, exposed a mass in the right parietal lobe suggestive of a primary tumor.

In the 1980s, MRI using gadolinium-based contrast agents (GBCAs) was introduced into clinical practice, improving the detection of cerebral lesions and disease characterization for many cerebral and vascular pathologies investigated with MRI [5]. Disease of the cerebral tissue frequently impairs the integrity of the blood-brain barrier allowing GBCAs to leak through and increase the signal of T1-weighted images compared to unaffected regions of the brain [6]. It is crucial to recognize that contrast imaging studies can be unspecific and misleading since multiple brain pathologies can disrupt the integrity of the brain-blood barrier causing contrast enhancement [7,8]. Furthermore, static contrast enhancement alone cannot discriminate between low-grade and high-grade tumors thus once a lesion is identified, resection or stereotactic biopsy is required for histologic diagnosis [5]. Biopsy is preferred to resection in those with a less common presentation such as young age for our athlete.

To date, GBM is one of the most difficult brain tumors to treat due to its high resistance to both radiation and chemotherapy. Even with this known resistance, both chemotherapy and radiation are integral components of treatment due to GBM’s high likelihood of recurrence within a 2-3 cm margin of the excision of the primary tumor [6,9]. Conventional treatment of GBM includes surgical resection followed by concurrent chemotherapy and radiation. Chemotherapy most commonly consists of TMZ along with radiation for five days every 28 days for a total of six cycles [10]. TMZ causes the formation of 6-methylguanine cellular defects, which leads to cell death directly through apoptosis and autophagy as well as limiting tumor growth by inducing cellular senescence. TMZ also makes cells more sensitive to radiation therapy aiding in increased rates of tumor cell death [10]. One of the anatomic barriers to the treatment of GBM is the blood-brain barrier, which makes many chemotherapy agents unusable due to their inability to penetrate this barrier. Therefore, some forms of treatment have been focused on going directly to the site of the tumor using agents such as carmustine in the form of a biodegradable disc that is left at the resection site following surgical removal of the mass [10]. Some other therapies that are used for recurrent GBM include lomustine, fotemustine, and bevacizumab. These are oral and IV treatments that can cross the blood-brain barrier [12]. These are important treatments as the rate of recurrence with GBM is up to 80% [9].

With currently utilized treatment options, survival rates remain very low with median survival following a diagnosis of GBM currently only 15 months [6]. Approximately 50% of patients survive to one year following diagnosis, and approximately 10% of patients are alive five years after diagnosis [3]. Favorable prognostic factors include younger age at diagnosis, lower histological grade, supratentorial and cerebellar tumor location, and tumor size less than 5 cm [1,3,6]. Certain molecular markers including promoter-methylated MGMT, isocitrate dehydrogenase (IDH) 1 mutation, and 1p/19q co-deletion are also associated with a more favorable prognosis particularly due to their susceptibility to chemotherapeutic agents [3].

As with many other fields, advances in therapy have been made since a better understanding of the molecular and genetic basis of glioblastoma as these enable the utilization of agents that are able to target specific signaling pathways during the cell’s lifecycle as well as genetic mutations. These therapies include immunotherapy, both active and passive therapies [10]. Passive therapies introduce an agent or cell that activates the patient’s immune system with an anticancer effect. An example of this is agents that stimulate the cytokine IL-2 in the immune system. Once IL-2 is stimulated, immune-regulating cells in the body such as natural killer cells and T cells are activated that can target and destroy cancer cells [10]. Additional methods that are being researched include the creation of tumor vaccines. One such vaccine takes proteins from the patient’s tumor and relies on the patient’s immune system to be activated by the vaccine to respond against tumor cells [10]. Additional adjunct therapies are being investigated such as a ketogenic diet. The main theory as to how this may be beneficial is the tumor itself relies on glucose as its main source of energy for growth. Ketogenic diets limit glucose in the diet by reducing carbohydrate intake and increasing fat intake [10]. 

Additional research into the cause of GBM is ongoing, with most recent advancements being made in the understanding of its pathogenesis. Specific genes that are responsible for the growth and triggering the formation of the tumor have been identified in addition to a better understanding of which gene expressions confer better or worse outcomes. Expression of the gene Tumor Protein, TP53, for instance, is associated with worse outcomes due to its responsibility in tumor cell malignancy, drug resistance, and metastases [13]. Identification of genes such as this will likely be pivotal for further advancements in the treatment of GBM. Another example is the B-RAF V600E mutation, which is a part of the pathway that regulates cell growth. For tumors in which it is present, this could be a potential target of therapies including kinase inhibitors as shown in some pediatric high-grade gliomas [13]. While the genomics is still under investigation at this time, we know that the overwhelming majority of glioblastoma diagnoses have no known familial predilection, and fewer than 1% of glioblastomas are attributed to genetic predisposition associated with diseases such as neurofibromatosis 1 and 2, retinoblastoma, tuberous sclerosis, Turcot’s syndrome, and Li-Fraumeni [3]. The only known risk factor that has shown definitive association with the development of brain tumors, including glioblastoma, is ionizing radiation [1,3]. Many efforts to identify specific occupational or environmental risk factors for the development of glioblastoma have proven inconclusive and underpowered [1]. 

The weight of a GBM diagnosis should not be underestimated. Given the low number of treatment options, the likelihood of recurrence, and unfavorable chances of survival to five years, it is not surprising that there are high rates of anxiety and depression among those with a GBM diagnosis [12]. While patients frequently feel supported by physicians, physical therapists, family members, and friends, they often request more well-rounded support systems in their care teams [12]. As reported by patients, dieticians, psychologists, pastors, and support groups are frequently requested in addition to wanting additional support from team members including physicians [12]. While dieticians are not always included as team members, up to 15% of patients requested wanting support from them. This is an important consultation to consider when caring for GBM patients, especially with new and ongoing research related to cancer diets [10,12]. Although psychologists are often included in patient care teams, one study reported that only 13% of patients felt supported by their psychologist [12]. This reinforces the importance of a well-rounded, multi-disciplinary team that can provide varying levels of support from the onset of diagnosis.

University athletes are in a unique scenario where they are frequently cared for by a multidisciplinary team at the university that includes medical, psychological, coaching, athletic training, nutrition, and conditioning team members from the start of their career as a student-athlete. Regardless of the diagnosis, this team remains available to the student-athlete so long as they are enrolled at the university. These team members also have the advantage of having known the athlete prior to the diagnosis and therefore have established bonds with them. This increases the level of support the student-athlete has, in addition to increasing the overall number of care team members once the student-athlete is established at a cancer institution. In the care of our student-athlete, it was also imperative that teammates, members of the athletic organization, and family members have access to personal psychological support and be educated on how to positively interact with the ill student-athlete. It has been demonstrated that caregivers frequently experience caregiver burden and request additional support services similar to those requested by the patient diagnosed with GBM [12]. At the university level, it is easy to connect the teammates and members of the athletic organization to these services that are a part of the university athletics care team.

While the diagnosis of GBM is unlikely in a collegiate athlete, maintaining a broad differential, including brain tumor, when a patient presents with symptoms similar to ours is important. If in the event a student-athlete does receive this diagnosis they have the advantage of previously being cared for by a multi-disciplinary team in the university setting that they can continue to receive care from during at least the initial stages of GBM treatment while they are still enrolled at the university. GBM continues to be a daunting diagnosis but with the advent of an improved understanding of the genomics of the tumor cells, hopefully, new treatment options are on the horizon.

## Conclusions

GBM is an aggressive form of cancer that affects the brain and spinal cord. Treatment of glioblastoma involves a multi-factorial approach with surgical resection, radiotherapy, and chemotherapy. Many patients benefit from cancer rehabilitation programs in which a multidisciplinary team manages physical, psychological, and social domains. When treating competitive athletes, it is recommended that they continue to receive support from their previously established university care team in addition to their newly established neuro-oncology team. With this diagnosis, it is imperative to provide personal psychological support and education to the athletic organization and teammates on how to positively interact with the ill athlete.
